# Comparative Analysis of Women With Notable Subjective Health Indicators Compared With Participants in the Australian Longitudinal Study on Women’s Health: Cross-Sectional Survey

**DOI:** 10.2196/publichealth.9490

**Published:** 2018-01-10

**Authors:** Christoph Schnelle, Eunice J Minford, Vanessa McHardy, Jane Keep

**Affiliations:** ^1^ School of Public Health Faculty of Medicine University of Queensland Herston, Queenland Australia; ^2^ Faculty of Medicine, Health and Life Sciences Queen's University Belfast Antrim Ireland; ^3^ Light Education Training Ltd London United Kingdom; ^4^ The Leaders Leader Greater London United Kingdom

**Keywords:** women’s health, health surveys, public health, Australian Longitudinal Study on Women’s Health, ALSWH, Universal Medicine, preventive medicine, health care costs, complementary therapies, cross-sectional studies

## Abstract

**Background:**

At least six communities with unusually good health and longevity have been identified, but their lifestyles aren’t adopted widely. Informal evidence suggests that women associated with Universal Medicine (UM), a complementary medicine health care organization in Eastern Australia and the United Kingdom with normal lifestyles, also have several unusual health indicators.

**Objective:**

Our objective was to determine how UM participants compared with women in the Australian population at large on a variety of health indicators.

**Methods:**

In an Internet survey conducted July to September 2015, a total of 449 female UM participants from 15 countries responded to 43 health indicator questions taken from the Australian Longitudinal Study on Women’s Health (ALSWH).

**Results:**

Survey responses revealed large positive differences in mental and physical health when compared with the ALSWH respondents, except for abnormal Pap test and low iron history. Differences and corresponding effect size estimates (Cohen *d*; ≥0.8 is a high difference, ≥0.5 a medium and ≥0.2 a small one with *P*<.001 except where indicated) included body mass index (BMI; 1.11), stress level (0.20, *P*=.006), depression (0.44), summary physical (0.31) and mental health (0.37), general mental health (0.39), emotional (0.15, *P*=.009) and social functioning (0.22), vitality (0.58), and general health (0.49), as well as lower incidences of diabetes, hypertension, and thrombosis (*P*<.001 each). Neither education levels nor country of residence had predictive value. Age did not predict BMI.

**Conclusions:**

The women’s responses notably claim substantially lower levels of illness and disease than in the general Australian population.

**Trial Registration:**

Australian New Zealand Clinical Trials Registry (ANZCTR): ACTRN12617000972325; https://www.anzctr. org.au/Trial/Registration/TrialReview.aspx?id=373120&isReview=true (Archived by WebCite at http://www.webcitation.org/ 6wEDDn45O)

**International Registered Report Identifier (IRRID):**

DERR2-10.2196/7993

## Introduction

The population in the developed world is getting both older and, even after accounting for age, less healthy. Increasing morbidity and the associated rise in health care costs [1-4], especially for diabetes and obesity [5-8], present serious and ever-growing societal challenges.

There have always been individuals who live exceptionally healthy lives, but entire communities that demonstrate superior health are rare; 4 such groups are the residents of the small US Pennsylvanian town of Roseto [9], Seventh Day Adventists [10], traditional Japanese [11], and senior Whitehall (UK) civil servants [12]. However, their lifestyles have not been adopted widely.

We report a survey of a group of people, primarily women, who form a community that is scattered throughout the world. The unifying characteristic is participation in an organization called Universal Medicine (UM), as described below. Informal evidence that can be gathered by visiting any UM-run event shows a low number of overweight or obese participants present, even though most events do not involve physical activity and few members profess to be on weight loss diets at any time. In addition, older participants seem not to have a higher body mass index (BMI) than younger participants.

## Methods

### Participants

Of all UM participants, 76% are female; therefore, for this initial investigation, we chose to focus on comparing the health of women in UM with that of a larger female population. For comparison, we adopted a substantial portion of questions from the Australian Longitudinal Study on Women’s Health (ALSWH). The ALSWH survey was inaugurated in 1996 and includes data from approximately 75,000 women [13-16].

We designed this study to answer the following questions. (1) Do women who participate in UM appear to have overall health substantially different from that of the general female population, as represented by ALSWH respondents? (2) In which specific aspects of health do UM participants differ, and in which aspects are there no differences? (3) Is UM participants’ health correlated with age, education, or their country of residence?

### Universal Medicine

UM is a complementary medicine health care organization founded in 1999 near Lismore, in Eastern Australia. UM has approximately 200 male and 500 female regular attendees at its workshops and conferences. We drew the study sample from these regular participants.

Information on the UM website [17] describes the organization as follows:

Universal Medicine is committed to providing Complementary Health & Healing Services that are Universal [sic] in their approach towards medicine and healing.

Through practical philosophies that inspire more self-caring and self-loving choices in daily life, Universal Medicine supports people to explore their overall well-being, the development of energetic awareness, and the depth they can bring to their quality of life and relationships.

Teachings are delivered in the form of lectures, talks, audios and treatments from Universal Medicine clinics. [It] regularly holds courses, workshops and retreats throughout Australia and internationally.

This survey is the first scientific investigation to look at the health of the UM participants.

UM has developed several treatment modalities. One such modality, called Esoteric Connective Tissue Therapy, is being studied in a randomized controlled trial on chronic low back pain [18]. These treatment modalities were used by 99.7% (367/368) of UM respondents in the 12 months prior to taking the survey.

Like other institutions that support self-empowerment of women, such as the family court, women’s shelters, and social services, UM has been the target of criticism in the form of “a vigorous, determined campaign” [19] by a small number of detractors. In contrast, at least 34 registered medical professionals are among the regular visitors to UM events. The curious coexistence of harsh critics, medical professional advocates, and anecdotal evidence of substantial benefit could make UM an interesting object of study.

### General Characteristics of the UM Survey and its Relationship to the ALSWH

The full UM survey comprised 43 questions from the ALSWH, as well as a newly developed menstrual attitudes questionnaire; the latter is not discussed in this paper. We chose the ALSWH items to maximize comparability of the 2 groups. For comparisons, we used the electronic ALSWH data books [20], which give frequencies, mean scores, and, in some cases, standard deviations.

The ALSWH website [21] states that the ALSWH is

...a longitudinal survey of over 58,000 women in three cohorts who were aged 18-23 (the 1973-1978 cohort), 45-50 (the 1946-1951 cohort), and 70-75 when surveys began in 1996....ALSWH assesses women’s physical and mental health, as well as psychosocial aspects of health (such as socio-demographic and lifestyle factors) and their use of health services.

An additional 17,000 participants were added after the definition was written.

### Study Population

Our study population of interest comprised 500 UM-participating women who, although they were consumers of complementary medical services, had profiles that differed in important ways from those of typical complementary medicine adherents [22]: UM participants, as recorded in this survey, did not have poorer health or a higher use of registered medical professionals (nor was their use substantially lower) than the ALSWH respondents. One difference is that UM women were more likely to be middle-aged.

### Design, Privacy of Data, and Recruitment

The portion of the data collection that was relevant to this study was a quantitative, cross-sectional online survey of women’s health. We did not collect any directly identifying data; however, we did collect indirectly identifying data such as partial medical history and age in years. Due to privacy considerations, portions of the data will not be available for inclusion in a public repository. We present age as a range, and we exclude the medical history data.

We recruited participants via 2 overlapping mailing lists of 650 and 350 UM members, and by distributing flyers at several UM-sponsored events in 2015.

Ethical approval was given by the University of Queensland School of Public Health Research Ethics Committee on June 23, 2015 (CS23062015). The first item on the survey explained the purpose of the survey and asked participants to either grant their consent or decline to do so. The study is registered with the Australian New Zealand Clinical Trials Registry (ACTRN12617000972325). Multimedia Appendix 1 shows a Strengthening the Reporting of Observational Studies in Epidemiology (STROBE) checklist.

Deidentified data will be made available from the corresponding author on reasonable request. Data will not include potential identifiers, as outlined by Hrynaszkiewicz et al [23]. Specifically, this means that age will be categorized into intervals, and a list of medical procedures undergone and major illnesses will be excluded. Regrettably, the data cannot be shared in a public repository, as with such a small group, it would be relatively easy to identify individuals even with the above measures taken.

### Implementation

The survey was completed online in July to September 2015, using the Survs survey platform (Enough Pepper Lda). In the case of duplicates, we removed 1 of them.

### Data Analysis

We conducted informal focus groups with UM-participating women to help us identify the ALSWH items that seemed most relevant for comparison in this preliminary study, and not to exceed an average of 75 minutes in response time. Demographic items related to age, BMI, menstrual status, education, and lifestyle questions about smoking, alcohol use, number of general practitioner visits, and number of UM event visits were included. In addition to the 36-Item Short Form Survey (SF-36) [24], other standardized scales we incorporated were the Center for Epidemiologic Studies Depression Scale (CES-D) [25,26], Perceived Control Scale [27,28], and an ALSWH-developed multi-item summed score for perceived stress [14,29]. A further 6 items solicited information about sleep quality, 47 past diagnoses of medical issues, and frequency of 24 physical symptoms in the previous 12 months, with response options being “often,” “sometimes,” “rarely,” and “never.”

Of the ALSWH questions, 27 were asked twice, once with reference to the present, and once with reference to the time of the respondent’s first attendance at a UM event.

#### Adjusting for Age and Cohort

The ALSWH age data from the data books [20] consisted of frequencies, means, and standard deviations. Individual data were not available. The ALSWH longitudinal study consists of surveys that are typically administered every 3 years, with numerous changes made to the items from one administration to the next. The results for 15 surveys of 3 age cohorts, born in 1921-1926, 1946-1951, and 1973-1978 have been published.

Because the ALSWH questions can change from one administration to the next, for each particular UM survey question, not all 15 ALSWH surveys included a comparable question.

We calculated the adjusted UM group responses for each variable by first excluding all UM respondents with an age that was not covered by an ALSWH survey. For example, in the question asking about past skin cancer diagnoses, we calculated the UM rate from only the 69 respondents aged 45 to 50 years and the 6 respondents aged 70 to 75 years.

The weighted ALSWH data are weighted by the frequency of UM respondents for that age group. For example, a question about any skin cancer diagnosis in the past was used in 2 ALSWH surveys: the first ALSWH survey of the 1946-1951 cohort with respondents aged 45 to 50 years, and the first ALSWH survey with respondents born between 1921 and 1926 and aged 70 to 75 years. There were 69 UM respondents aged 45 to 50 years and 6 UM group respondents aged 70 to 75 years who gave valid responses; hence, the frequency weights used to calculate the weighted ALSWH skin cancer percentage (11.9%) were 69 and 6. This makes the UM results comparable with the ALSWH weighted results in terms of age.

#### Adjusting for Education

Age was a major predictor of the responses to many questions, but education turned out to have no predictive value. Hence, we did not adjust the data for education.

### Effect Size Calculations

We calculated Cohen *d* using the Stata command esizei (version 14.2; StataCorp LLC). The ALSWH standard deviations are reported to only 1 significant digit, so we assumed the maximum possible standard deviation (eg, 0.1 became 0.149 and 0.0 became 0.049, reducing Cohen *d*), from which we used the pooled standard deviation [30] with Welch’s approximation.

A superior approach for calculating *P* values and effect sizes would be to compare the UM respondents with their closest ALSWH counterparts, taking account of age, education, and BMI. When the full ALSWH data are available, we will take this approach.

## Results

### Survey Responses

**Figure 1:**
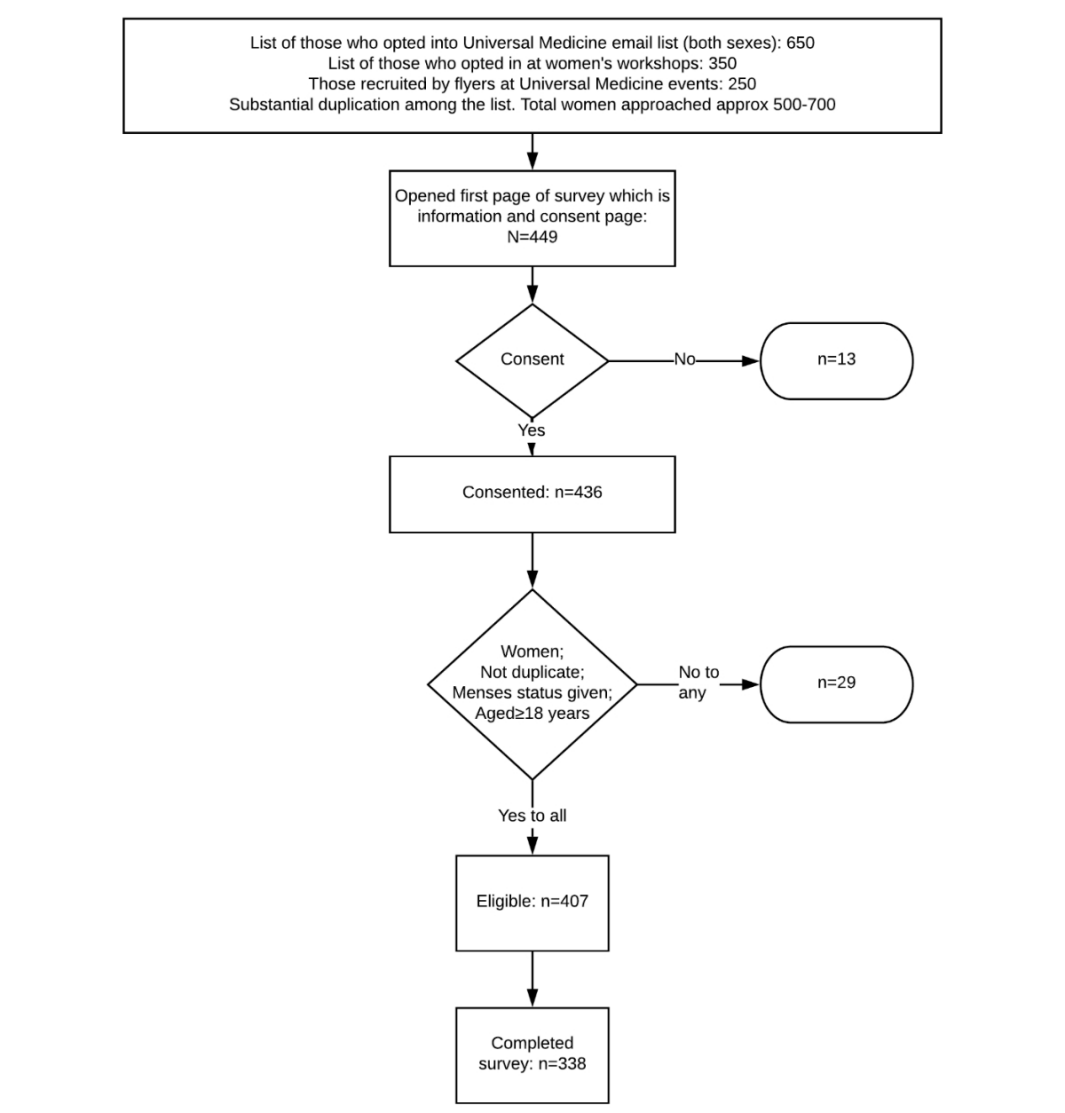
Recruitment flowchart for participants in the Universal Medicine survey.

This survey produced 449 responses, of which 373 (83.1%) were complete. The respondents answered from 17 countries (Australia, n=273; United Kingdom, n=97; Germany, n=26; the Netherlands, n=11; other European countries, n=18; United States, n=11; rest of the world, n=13). Of the respondents, 13 did not consent, 3 were male, 20 did not give their menses status, 5 were less than 18 years of age, and 1 completed the survey twice, leaving 407 valid and 338 completed responses (Figure 1).

Table 1 and Table 2 show demographic and survey administration data. The UM group was more highly educated than the ALSWH cohort. The average UM group survey respondent’s age was 48 (range 18-86) years; age was normally distributed (Shapiro-Wilk *P*=.42). The proportion of smokers among UM women was 1.6% (4/240), compared with 13.9% among ALSWH women. The rate of alcohol use among UM women was 1.8% (6/338) versus 86.3% for ALSWH women.

**Table 1:** Demographic and survey administration data for Universal Medicine (UM) participants compared with data from the Australian Longitudinal Study on Women’s Health (ALSWH).

Characteristics		UM cohort n (%)	ALSWH frequency weighted^a^	*P* value
**Menstrual status**				
	Total^b^	407		
	Menses	173 (42.5)		
	Perimenopausal	75 (18.4)		
	Menopausal	159 (39.1)		
**Educational attainment**				<.001
	Total^b^	193		
	No formal education	12 (6.2)	11.4	
	School certificate	3 (1.6)	21.2	
	High school	21 (10.9)	19.4	
	Trade, certificate, or diploma	71 (36.8)	20.9	
	Degree	66 (34.2)	17.3	
	Higher degree	20 (10.4)	9.8	
**Number of UM events attended per year by survey participants**				
	0-1	1 (0.25)		
	2-5	54 (13.3)		
	6-10	81 (19.9)		
	≥11	271 (66.6)		
**General practitioner visits**				<.001
	Total^b^	275		
	None	27 (9.8)	6.6	
	1-2	134 (48.7)	33.6	
	3-4	63 (22.9)	28.6	
	5-6	31 (11.3)	15.9	
	≥7	20 (7.3)	15.3	
**Smoking**				<.001
	Total^b^	240		
	Not at all	236 (98.3)	86.2	
	Less than weekly	2 (0.8)	1.7	
	Weekly	0 (0.0)	1.2	
	Daily	2 (0.8)	11.0	
**Alcohol consumption (drinks)**				<.001
	Total^b^	338		
	Daily	1 (0.3)	6.5	
	1-6/week	0 (0.0)	36.8	
	<1/week	5 (1.5)	42.8	
	Not for a year	38 (11.2)		
	Not for 5 years	294 (87.0)		
	Never		13.7	

^a^ALSWH percentages with UM group frequency weights.

^b^Number of UM respondents with ages that were surveyed in ALSWH for this particular question. Example: skin cancer was covered in ALSWH Mid 1 (45-50 years old) and Old 1 (73-78 years old) surveys; UM group had 69 respondents aged 45-50 years and 6 aged 73-78 years who gave valid responses; 4 of the 69+6=75 had skin cancer.

**Table 2:** Continuous demographic variables for Universal Medicine (UM) participants.

Continuous variables	n	Mean	SD^a^	Median	Range		Interquartile range	
					Minimum	Maximum	25%	75%
Age (years)	407	47.7	11.8	47	18	86	40	56
Years with UM	392	7.9	3.3	8.0	0.5	15.6	5.2	10.1
Body mass index (kg/m^2^)	323	21.0	3.0	20.6	15.2	39.0	19.1	22.4
Age at menopause (years)	137	49.9	5.1	50	27	58	47	52
Age at menstruation (years)	338	13.1	1.6	13	9	19	12	14

^a^SD: standard deviation.

**Figure 2:**
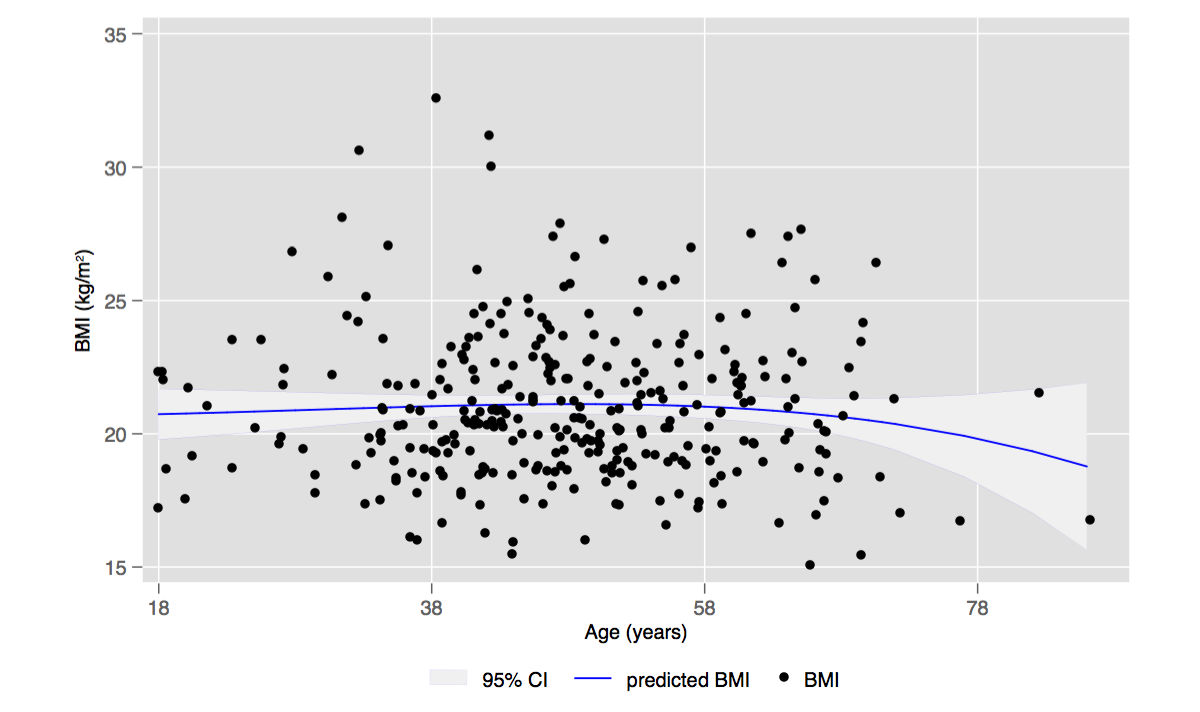
Distribution of body mass index (BMI) by age of participants using fractional polynomial line of best fit including 95% CI. Note that the line is almost straight; that is, there is almost no association between BMI and age. In the general population, BMI rises with age up to 69 years (source: Australian Bureau of Statistics).

The UM respondents’ average BMI of 21.0 kg/m^2^ was substantially lower than the ALSWH weighted BMI of 26.1 kg/m^2^ (see Table 3). A regression analysis showed that the BMI for the UM respondents was related to neither education (*P*=.63) nor age (*P*=.56), in contrast to data from the ALSWH and the Australian Bureau of Statistics showing obesity rates increasing to ages 55-64 (men) and 65-74 (women) [31]. A fractional polynomial graph (a visual means for determining whether a relationship is linear or, for example, U-shaped) of age and BMI shows an almost straight horizontal line; that is, there is no apparent relationship (Figure 2).

Table 3 shows the lower BMI scores for UM respondents. UM responses scored higher (better) on the 4 common psychological health scales that measure stress, the level of perceived control, depression, and the mental and physical health scales of the SF-36.

Table 4 compares the proportion of UM respondents who reported ever having had a particular health issue (including the time before they became associated with UM) with weighted ALSWH responses. The UM respondents had substantially higher rates than the ALSWH group of abnormal Pap tests and diagnoses of low iron, and substantially lower rates of diabetes, hypertension, and thrombosis.

*P* values use Pearson chi-square for the hypothesis that the rows and columns in the 2-way listing of UM respondents and ALSWH respondents in Table 4 are independent.

UM respondents had fewer sleep issues than did ALSWH respondents, with 68.9% (228/331) of the former and 27.9% of the latter reporting no issues at all. For lying awake most of the night, the percentages were 2.4% (UM, 8/331) versus 14.2% (ALSWH); taking a long time to get to sleep (38/331, 11.5% vs 28.8%), being kept awake by worrying (28/331, 8.5% vs 18.9%), and sleeping badly at night (44/331, 13.3% vs 35.2%) (Multimedia Appendix 2).

**Figure 3:**
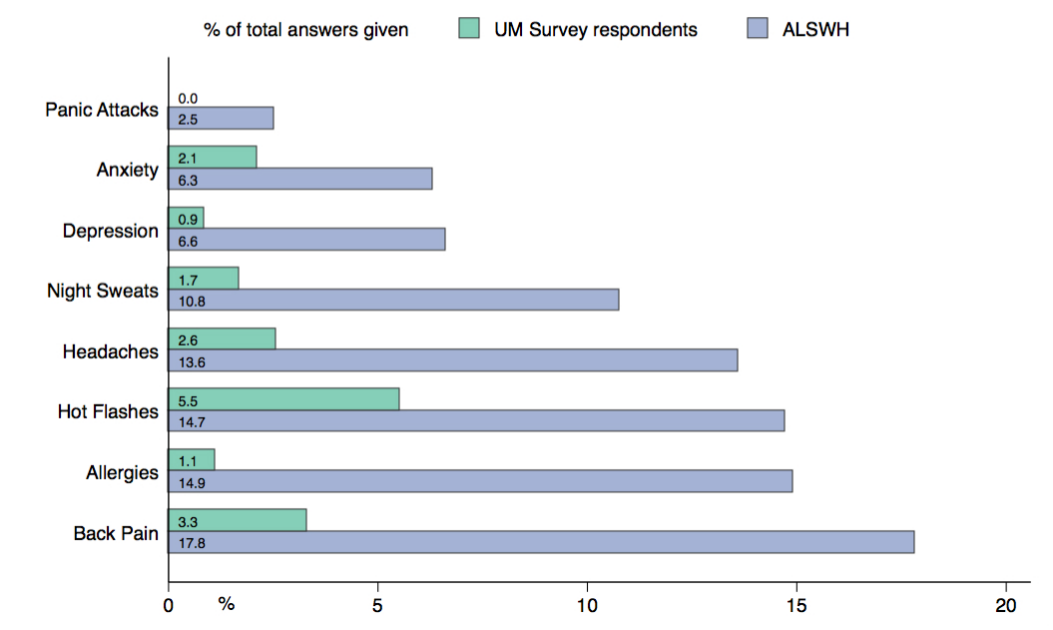
Differences in symptoms between women participating in Universal Medicine (UM) and respondents to the Australian Longitudinal Study on Women’s Health (ALSWH). All *P* values <.001.

Multimedia Appendix 3 shows the symptoms for which the UM respondents were noticeably different (in all cases noticeably fewer incidences) from their ALSWH counterparts. The lower rates of back pain, allergies, breathing difficulties, depression, panic attacks, headaches, migraines, hot flashes, and night sweats could be of interest (Figure 3).

We detected no difference of any substance for an effect of education, including BMI, Stress Scale, Perceived Control Scale, CES-D, and SF-36. Multimedia Appendix 4 shows this in graphical form.

**Figure 4:**
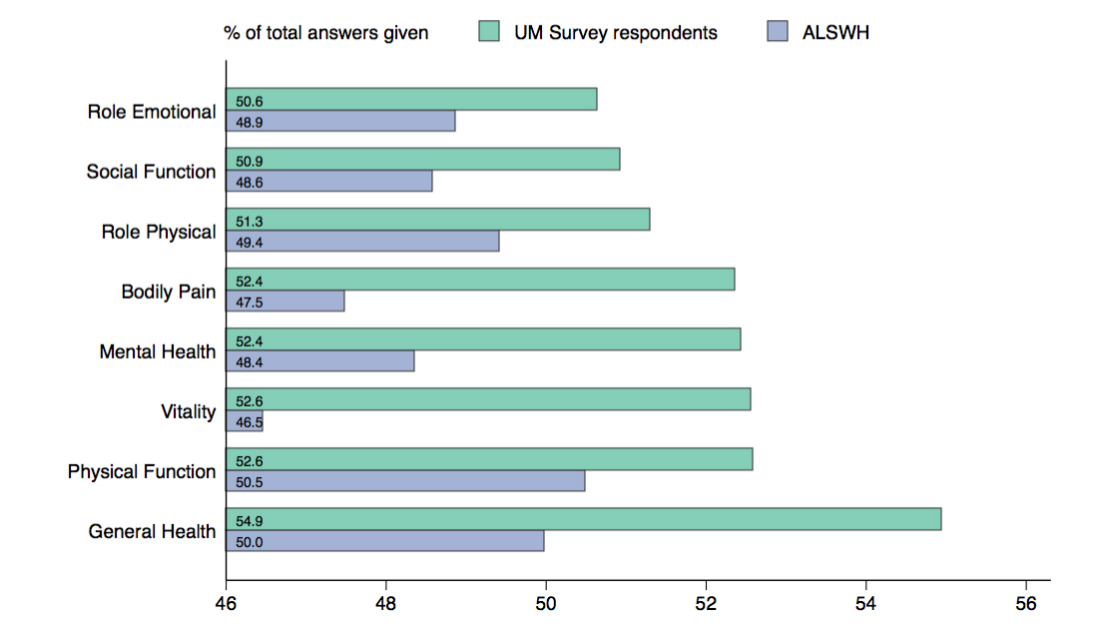
Differences in composite health indicators (36-Item Short Form Survey, SF-36) between women participating in Universal Medicine (UM) and respondents to the Australian Longitudinal Study on Women’s Health (ALSWH). Weighted responses; all scores are normalized to mean 50, standard deviation 10.

UM survey responses scored more highly than ALSWH responses on composite physical and mental health (see Figure 4). For ALSWH respondents, physical scores peaked at age 18 to 23 years and mental scores peaked at age 73 to 78 years. UM survey respondents’ scores were similar to peak ALSWH scores; that is, UM respondents had the higher physical scores of the very young and the higher mental scores of the very old.

### Effect Sizes

Observed effect sizes (Cohen *d*) ranged from 0.6 to 11.9 (Table 3). These values are with one exception higher than the 0.8 considered to denote a large effect [33,34].

### Linking UM Participation to Outcomes: Preliminary Exploration

A cross-sectional survey is not able to establish causality, so it is possible that participants for whom UM events held appeal were already healthier. However, there are indications in the data that this was not the case. The UM survey asked respondents to answer 27 of the 43 ALSWH questions as per their memory of how they were feeling at the time of their first UM event and to answer the same questions in relation to the present. Multimedia Appendix 2 shows BMI changes. Retrospective reports are always subject to the vagaries of memory and have low reliability, but until a prospective longitudinal study is undertaken, these data are the best available. The data indicate the UM respondents were of average or below-average physical health and below-average mental health at the time of their first UM event. Examples are the proportion who often had back pain in the previous 12 months: 25.8% (UM past, 87/337) versus 3.3% (UM present, 11/336) versus 17.8% (ALSWH); those who had depression: 18.3% (61/333) versus 0.6% (2/332) versus 6.6%; and those who had allergies: 26.0% (86/331) versus 0.9% (3/332) versus 14.9%.

**Table 3:** Results from standard survey scales in the Australian Longitudinal Study on Women’s Health (ALSWH) and Universal Medicine (UM) groups, with *r* values and standard deviation.

		Survey respondents with ages covered by ALSWH surveys				ALSWH respondents with UM frequency weights			Effect size			
		n^a^	Mean	95% CI	SD^b^	Mean	95% CI	SD^b^	Cohen	95% CI	*r* ^c^	*pc*
Body mass index (kg/m^2^)		253	21.0	20.7-21.4	2.97	26.1	25.9-26.2	4.6	1.11	0.98-1.23	.48	4*10^-66^
Stress^d^ (lower is better)		200	0.63	0.55-0.70	0.52	0.73	0.72-0.75	0.53	0.20	0.057-0.38	.10	0.0059
Perceived Control Scale^d^		135	4.9	4.8-5.0	0.63	4.3	4.3-4.3	0.79	0.74	0.57-0.91	.35	2*10^-17^
CES-D^d^ (lower is better)		233	3.6	3.1-4.2	5.60	6.1	6.1-6.2	5.6	0.44	0.31-0.57	.21	6*10^-11^
**SF-36^d^**												
	Summary Physical Health	272	52.8	51.9-53.6	10.0	49.7	49.4-49.9	10.0	0.31	0.19-0.43	.15	6*10^-7^
	Summary Mental Health	272	51.4	50.4-52.5	10.0	47.7	47.5-47.9	10.0	0.37	0.25-0.50	.18	10^-9^
	General Mental Health	295	80.1	78.5-81.7	13.6	73.2	72.9-73.4	17.9	0.39	0.27-0.51	.19	5*10^-11^
	Role Emotional	294	85.3	82.2-88.3	26.5	79.6	79.2-79.9	36.9	0.15	0.038-0.27	.08	0.0091
	Social Functioning	295	87.1	84.9-89.3	19.1	81.9	81.7-82.1	24.0	0.22	0.10-0.33	.11	0.0002
	Vitality	295	69.5	67.6-71.5	17.2	57.5	57.2-57.8	20.7	0.58	0.47-0.70	.28	9*10^-23^
	General Health	275	81.9	80.0-83.8	15.9	71.8	71.6-71.9	20.9	0.49	0.36-0.61	.24	3*10^-15^
	Bodily Pain	294	82.8	80.6-85.0	19.5	70.7	70.4-70.9	24.0	0.51	0.39-0.62	.25	2*10^-17^
	Role Physical	294	84.8	81.6-88.0	27.9	78.2	77.9-78.6	36.2	0.18	0.10-0.34	.09	0.0019
	Physical Function	294	89.5	87.9-91.0	13.3	84.6	84.1-85.1	19.7	0.25	0.16-0.40	.12	0.00003

^a^Number of UM respondents with ages that were surveyed in ALSWH for this particular question.

^b^SD: standard deviation.

^c^The r value was calculated as r=d /(sqrt[4+ d^2^]), where *d* is Cohen *d* as derived from the formula given by Nakagawa and Cuthill [32]. *P* value calculated with Satterthwaite’s *t* test.

^d^Multi-item summed scores for perceived stress, Perceived Control Scale, Center for Epidemiologic Studies Depression Scale (CES-D), and 36-Item Short Form Survey (SF-36) using Australian coefficients.

**Table 4:** Reported diagnoses among Universal Medicine (UM) and Australian Longitudinal Study on Women’s Health (ALSWH) respondents.

Have you ever been diagnosed with:		UM response “yes” (ALSWH ages^a^)		ALSWH^b^ weighted (%)	*P* value
		n	%		
**UM worse than ALSWH**					
	Abnormal Pap test	73	35.3	22.2	<.001
	Low iron	38	41.3	29.8	.02
**No significant difference**					
	Abnormal mammogram	38	22.0	17.8	.16
	Asthma	13	15.1	16.6	.71
	Bronchitis or emphysema	14	18.7	18.9	.96
	Breast cancer	2	2.7	2.4	.88
	Cervical cancer	3	4.0	3.1	.66
	Heart disease	2	2.2	2.8	.72
	Osteoporosis	6	8.0	5.6	.37
	Skin cancer	4	5.3	11.9	.08
	Stroke	0	0.0	1.2	.34
**UM better than ALSWH**					
	Diabetes	0	0.0	3.1	<.001
	Hypertension	0	0.0	19.2	<.001
	Thrombosis	2	0.7	4.5	<.001

^a^Percentage of UM respondents with ages that were surveyed in ALSWH for this particular question. Example: skin cancer was covered in ALSWH Mid 1 (45-50 years old) and Old 1 (73-78 years old) surveys; UM group had 69 respondents aged 45-50 years and 6 aged 73-78 years who gave valid responses; 4 of the 69+6=75 had skin cancer.

^b^ALSWH percentages with UM group frequency weights.

## Discussion

The results from this survey suggest that on several health indicators this group of women was faring notably differently from, and from looking at the data, better than, the general population, as represented by the ALSWH respondents. UM respondents differed in a variety of aspects of physical and mental health, except for lifetime diagnoses of positive Pap smears and low iron. However, these results should be interpreted with caution, as a cross-sectional survey is not able to establish causality.

### Principal Findings

The results show that the UM participants, who on average were 48 years old and had been associated with UM for 8 years, had a lower BMI than the general population; experienced substantially less frequent back pain, lower stress, and depression scores; and scored higher on general mental and physical health, vitality, and perceived level of control. UM participants also had noticeably lower lifetime diagnoses of hypertension, used less hormone replacement therapy, had fewer sleep issues, and had notably fewer instances of allergies, sinusitis, anxiety, breathing difficulties, panic attacks, headaches, migraines, hot flashes, and night sweats. Major differences in lifestyle choices were apparent between the UM participants and the ALSWH population, with very low rates of both alcohol use and smoking in the UM group.

There are other groups that demonstrate aspects of above-average health. What may make it worthwhile to investigate the UM group further is the breadth of health indicators for which their scores are better than those of the general population.

### About Universal Medicine

If it is the case that UM participants are healthier than their population cohorts, and that they were not already healthier when they began participating in UM, this raises the question of whether UM practices or interventions could be responsible for this difference and whether there is any plausible mechanism that could explain UM’s effects, if any.

The organization’s founder, Serge Benhayon, has described UM as encompassing the concept:

...that [there] is medicine in responsibility, that there’s medicine in caring, that there’s medicine in nurturing. There is great medicine in love, there is great medicine in stillness, great medicine in harmony, great medicine in surrender, tenderness, preciousness, that there is medicine in everything. Everything is, in fact, eventually medicine, or bad medicine, if we abuse it.

### Study Limitations

One limitation of the study is that only summary ALSWH data were available for comparison. This precluded a case-control analysis, wherein UM respondents would be individually matched to ALSWH respondents. A second limitation is that the UM sample was self-selected and much smaller than the ALSWH group, although 449 respondents constitutes a large majority of the estimated 500 to 600 eligible UM event visitors (women in 2015 who had participated in at least one UM event). While this study is not able to make causal inferences about UM, it might have served to inform identification of promising variables to include in future studies. A third limitation is the possibility of response bias or memory bias among UM respondents. A fourth limitation is that 17% of UM respondents dropped out during the survey; however, the dropout population did not differ significantly in their health outcomes from the rest of the respondents.

### Future Research

A future study using regression analysis to uncover the association of lifestyle choices and demographics with physical and mental health may be of value. As mentioned previously, a true longitudinal, controlled study of UM participants would be worthwhile, as it would give a more accurate picture of participants’ initial health levels, the type and level of specific UM services they access, and the trajectory of their health changes. Such investigation of dose-response relationships and causal factors could inform development of lifestyle modification programs that individuals could use in concert with medicine.

### Conclusions

UM-participating women appear to be notable in that they exhibit better than average health and are not members as a result of any competitive selection, unlike senior Whitehall public servants, who are selected on a competitive basis; nor are UM-participating women part of a population with a limiting cultural or religious tradition. UM women also come from a wide range of backgrounds, ages, and countries. Identifying a group of women who are not part of the obesity epidemic and measuring their other health indicators may be of use for further research. Therefore, further research on this group of women could be worthwhile.

## Appendix

Multimedia Appendix 1 Strengthening the Reporting of Observational Studies in Epidemiology (STROBE) checklist.

Multimedia Appendix 2 Sleep issues, BMI changes, and BMI changes by age.

Multimedia Appendix 3 Symptoms for which Universal Medicine (UM) respondents fared better than Australian Longitudinal Study on Women’s Health (ALSWH) respondents.

Multimedia Appendix 4 Lack of association between education and other scales.

## Abbreviations

ALSWH: Australian Longitudinal Study on Women’s Health
BMI: body mass index
CES-D: Center for Epidemiologic Studies Depression Scale
SD: standard deviation
SF-36: 36-Item Short Form Survey
STROBE: Strengthening the Reporting of Observational Studies in Epidemiology
UM: Universal Medicine
