# Supplementary material for: Comparative Analysis of Women With Notable Subjective Health Indicators Compared With Participants in the Australian Longitudinal Study on Women’s Health: Cross-Sectional Survey
Source: JMIR Public Health Surveill. 2018 Jan 10;4(1):e6. doi: 10.2196/publichealth.9490 (PMC5784184; doi:10.2196/publichealth.9490)
Supplement: Multimedia Appendix 2 [file publichealth_v4i1e6_app2.pdf]

Sleep issues in graphical form

Showing this quote from the paper in graphical form:

"UM respondents had fewer sleep issues than did ALSWH respondents, with 68.9% (228/331) of the former and 27.9% of the latter reporting no issues at all. For lying awake most of the night, the percentages were 2.4% (UM, 8/331) versus 14.2% (ALSWH); taking a long time to get to sleep (11.5% 38/331 vs 28.8%), being kept awake by worrying (8.5% 28/331 vs 18.9%), and sleeping badly at night (13.3% 44/331 vs 35.2%)."

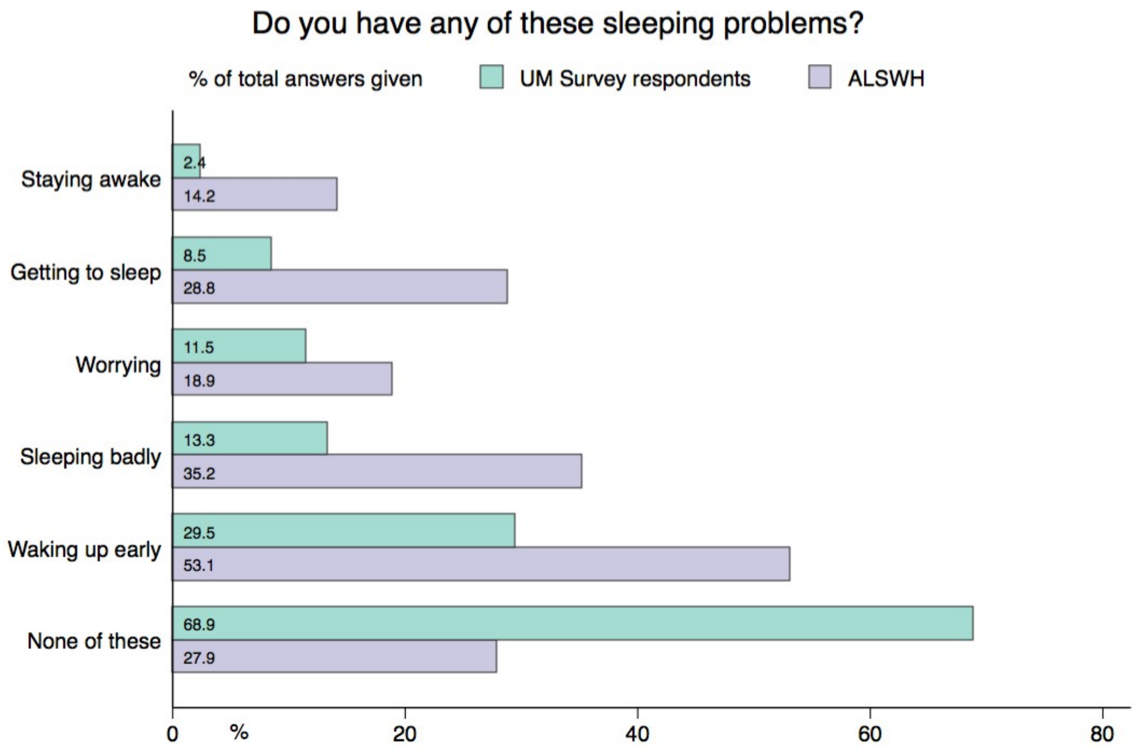

UM: Universal Medicine

Changes in BMI

UM respondents were asked to provide their weight as it was at the time of their first UM event. They were also asked to provide their weight as of at the time of taking the survey. This graph shows the difference in BMI between the two measurements over the past BMI. According to the respondents, on a population level, the higher the initial BMI, the higher the average weight loss.

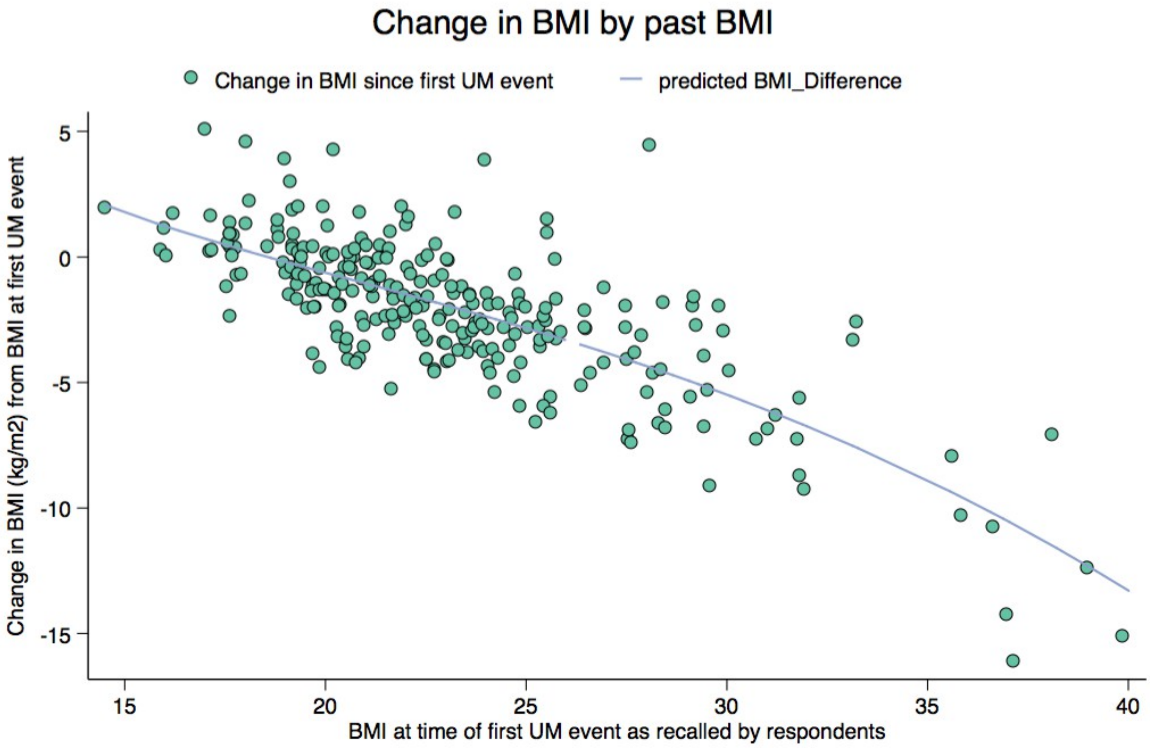

UM: Universal Medicine

Both x-axis and y-axis are measured in BMI (kg/m^2). For example, for the mean height given in the survey by the respondents of 1.66m, each BMI point represents 2.76kg. The mean weight change reported was -2.23 BMI points. Of the respondents, 18% (47/266) reported a gain in weight, 6.8% (18/266) no change, and 76% (201/266) a reduction in weight.

As the past weight data is subject to the vagaries of memory, the above graph should be taken with caution and only as an indication what the UM survey participants remember.

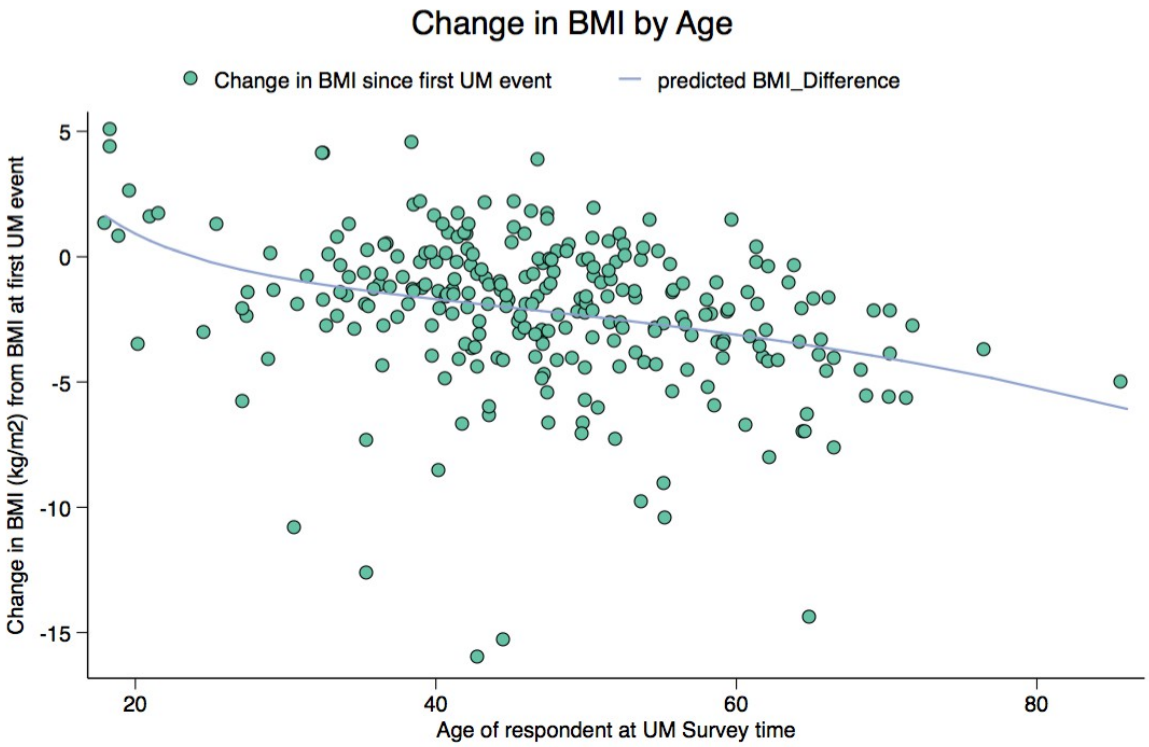

UM: Universal Medicine

In the general population, BMI increases with age. As BMI is not associated with age among UM Survey respondents, older UM participants would be expected to have lost more weight on average than younger UM participants. This is borne out in the graph above, though this graph is just as much subject to memory issues as the previous graph.
