# Supplementary material for: Comparative Analysis of Women With Notable Subjective Health Indicators Compared With Participants in the Australian Longitudinal Study on Women’s Health: Cross-Sectional Survey
Source: JMIR Public Health Surveill. 2018 Jan 10;4(1):e6. doi: 10.2196/publichealth.9490 (PMC5784184; doi:10.2196/publichealth.9490)
Supplement: Multimedia Appendix 3 [file publichealth_v4i1e6_app3.pdf]

Multimedia Appendix 3. Comparing the responses from Universal Medicine (UM) and Australian Longitudinal Study on Women's Health (ALSWH) participants to a question asking how often they experienced these symptoms in the past.

ALSWH Symptoms<br>**Symptoms**		UM unweighted N	UM unweighted %	UM weighted N	UM weighted %	ALSWH %
**Allergies hayfever sinusitis**						
	Never	220	66.3	177	65.8	40.0
p<0.0005 (*2*10^{-27}*)	Rarely	68	20.5	54	20.1	17.3
UM weighted N total 333	Sometimes	41	12.3	35	13.0	27.8
	**Often**	3	0.9	3	**1.1**	**14.9**
**Anxiety**						
	Never	113	33.4	52	36.4	46.8
p<0.0005 (*3*10^{-12}*)	Rarely	140	41.4	56	39.2	24.2
UM weighted N total 175	Sometimes	75	22.2	32	22.4	22.7
	**Often**	10	3	3	**2.1**	**6.3**
**Back pain**						
	Never	108	32.1	86	31.6	25.7
p<0.0005 (*9*10^{-5}*)	Rarely	139	41.4	112	41.2	23.0
UM weighted N total 333	Sometimes	78	23.2	65	23.9	33.6
	**Often**	11	3.3	9	**3.3**	**17.8**
**Breathing difficulty**						
	Never	273	82.7	204	82.9	71.7
p<0.0005 (*3*10^{-6}*)	Rarely	41	12.4	33	13.4	13.4
UM weighted N total 306	Sometimes	15	4.5	8	3.3	12.0
	**Often**	1	0.3	1	**0.41**	**2.9**
**Depression**						
	Never	258	77.7	183	77.5	56.6
p<0.0005 (*1*10^{-14}*)	Rarely	49	14.8	37	15.7	18.6
UM weighted N total 293	Sometimes	23	6.9	14	5.9	18.2
	**Often**	2	0.6	2	**0.85**	**6.6**
**Episodes of intense anxiety eg panic attacks**						
	Never	297	90	179	89.1	75.1
p<0.0005 (*2*10^{-9}*)	Rarely	28	8.5	19	9.5	13.6
UM weighted N total 249	Sometimes	5	1.5	3	1.5	8.8
	**Often**	0	0	0	**0.0**	**2.5**

ALSWH Symptoms<br>**Symptoms**		UM unweighted N	UM unweighted %	UM weighted N	UM weighted %	ALSWH %
**Eyesight problems**						
	Never	183	55.5	70	37.2	35.2
p<0.0005, p=0.001 (weighted)	Rarely	72	21.8	56	29.8	19.9
UM weighted N total 238	Sometimes	61	18.5	50	26.6	32.3
	**Often**	14	4.2	12	**6.4**	**12.7**
**Headaches, migraines**						
	Never	113	33.6	91	33.3	17.3
p<0.0005 ($3*10^{-31}$)	Rarely	160	47.6	127	46.5	31.3
UM weighted N total 333	Sometimes	55	16.4	48	17.6	37.7
	**Often**	8	2.4	7	**2.6**	**13.6**
**Hot flashes**						
	Never	245	74.2	113	62.4	44.5
p<0.0005 ($9*10^{-27}$)	Rarely	45	13.6	35	19.3	16.2
UM weighted N total 230	Sometimes	29	8.8	23	12.7	24.6
	**Often**	11	3.3	10	**5.5**	**14.7**
**Indigestion, heartburn**						
	Never	225	68	166	66.9	48.2
p<0.0005 ($3*10^{-12}$)	Rarely	66	19.9	52	21.0	22.2
UM weighted N total 293	Sometimes	33	10	25	10.1	20.6
	**Often**	7	2.1	5	**2.0**	**7.8**
**Leaking urine**						
	Never	262	79.2	211	78.7	62.7
p<0.0005 ($3*10^{-9}$)	Rarely	43	13	34	12.7	16.3
UM weighted N total 333	Sometimes	20	6	17	6.3	15.8
	**Often**	6	1.8	6	**2.2**	**5.2**
**Night sweats**						
	Never	227	69.2	115	64.3	54.8
p<0.0005 ($4*10^{-11}$)	Rarely	58	17.7	36	20.1	15.0
UM weighted N total 230	Sometimes	40	12.2	25	14.0	19.4
	**Often**	3	0.9	3	**1.7**	**10.8**

ALSWH Symptoms<br>**Symptoms**		UM unweighted N	UM unweighted %	UM weighted N	UM weighted %	ALSWH %
**Palpitations feeling that your heart is racing or fluttering in your chest**						
	Never	216	64.9	110	64.3	61.2
$p<0.0005$ ($7*10^{-8}$)	Rarely	93	27.9	51	29.8	19.7
UM weighted N total 210	Sometimes	22	6.6	8	4.7	15.9
	**Often**	2	0.6	2	**1.2**	**3.2**
**Severe tiredness**						
	Never	96	28.4	67	27.7	28.5
$p<0.0005$ ($6*10^{-12}$)	Rarely	133	39.3	94	38.8	24.1
UM weighted N total 294	Sometimes	92	27.2	70	28.9	32.4
	**Often**	17	5	11	**4.6**	**15.1**
**Stiff or painful joints**						
	Never	153	45.9	117	45.2	30.4
$p<0.0005$ ($5*10^{-23}$)	Rarely	107	32.1	80	30.9	19.7
UM weighted N total 320	Sometimes	61	18.3	51	19.7	31.3
	**Often**	12	3.6	11	**4.3**	**18.7**
**Urine that burns or stings**						
	Never	291	88.4	236	88.7	77.9
$p<0.0005$ ($8*10^{-5}$)	Rarely	28	8.5	23	8.7	14.4
UM weighted N total 333	Sometimes	9	2.7	6	2.3	6.8
	**Often**	1	0.3	1	**0.38**	**1.0**
**Vaginal irritation or unusual discharge**						
	Never	258	77.5	209	77.7	68.3
$p=0.001$, $p=0.002$ (weighted)	Rarely	50	15	41	15.2	18.7
UM weighted N total 333	Sometimes	17	5.1	12	4.5	10.7
	**Often**	8	2.4	7	**2.6**	**2.3**
