# Supplementary material for: Comparative Analysis of Women With Notable Subjective Health Indicators Compared With Participants in the Australian Longitudinal Study on Women’s Health: Cross-Sectional Survey
Source: JMIR Public Health Surveill. 2018 Jan 10;4(1):e6. doi: 10.2196/publichealth.9490 (PMC5784184; doi:10.2196/publichealth.9490)
Supplement: Multimedia Appendix 4 [file publichealth_v4i1e6_app4.pdf]

Lack of association between education other scales

These graphs show the following quote from the paper in graphical form:

"We detected no difference of any substance for an effect of education, including BMI, Stress Scale, Perceived Control Scale, CES-D, and SF-36."

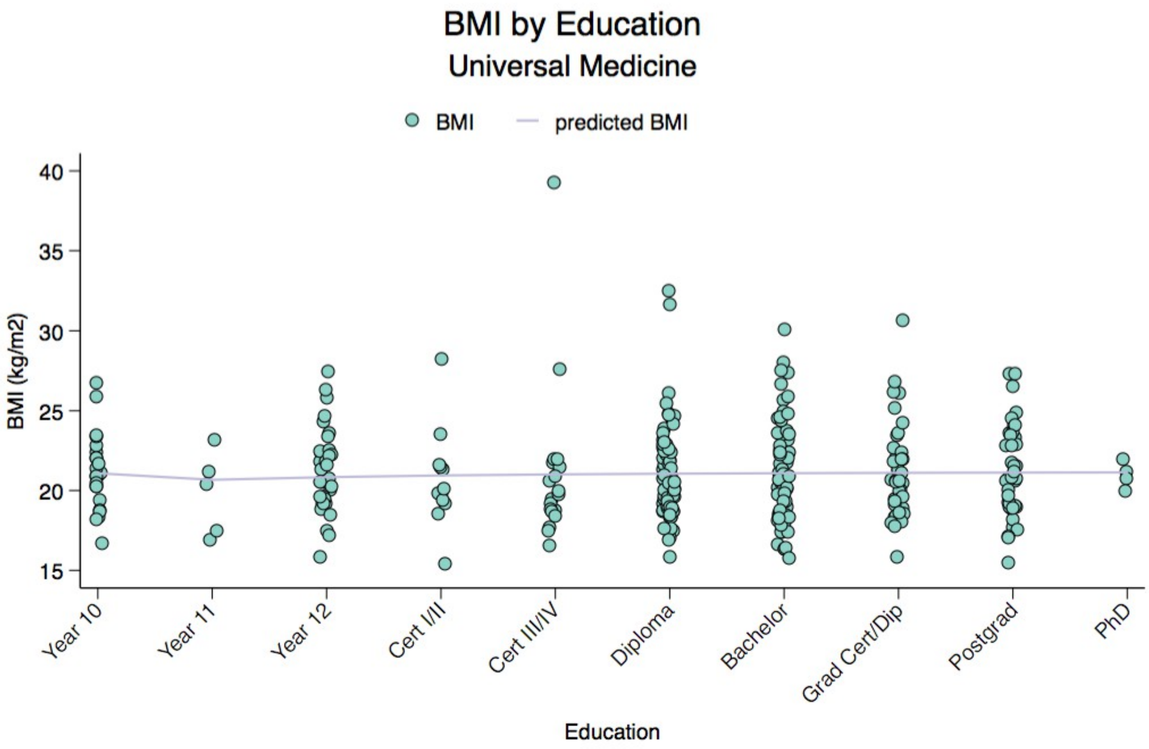

Line of best fit (fractional polynomial best fit) is almost completely flat, no association between BMI and Education in this graph and the graphs below. These UM survey results are unusual as higher education is usually an indicator of increased physical and mental well-being as summarised here: https://s3.amazonaws.com/academia.edu.documents/42790312/cutler.pdf?AWSAccessKeyId=AKIAIWOWYYGZ2Y53UL3A&Expires=1515264563&Signature=MGA7xfuwp6EeoxNYKSKDQbOZ41U%3D&response-content-disposition=inline%3B%20filename%3DEducation_and_Health_Evaluating_Theories.pdf (1259 references in google scholar. The title is "Education and health: evaluating theories and evidence" by David Cutler and Adriana Lleras-Muney.)

Educating people is an accepted way to improve physical and mental well-being. If UM has had any influence on the well-being of the UM Survey respondents, then this influence did not seem to be dependent on education, which seems unusual.

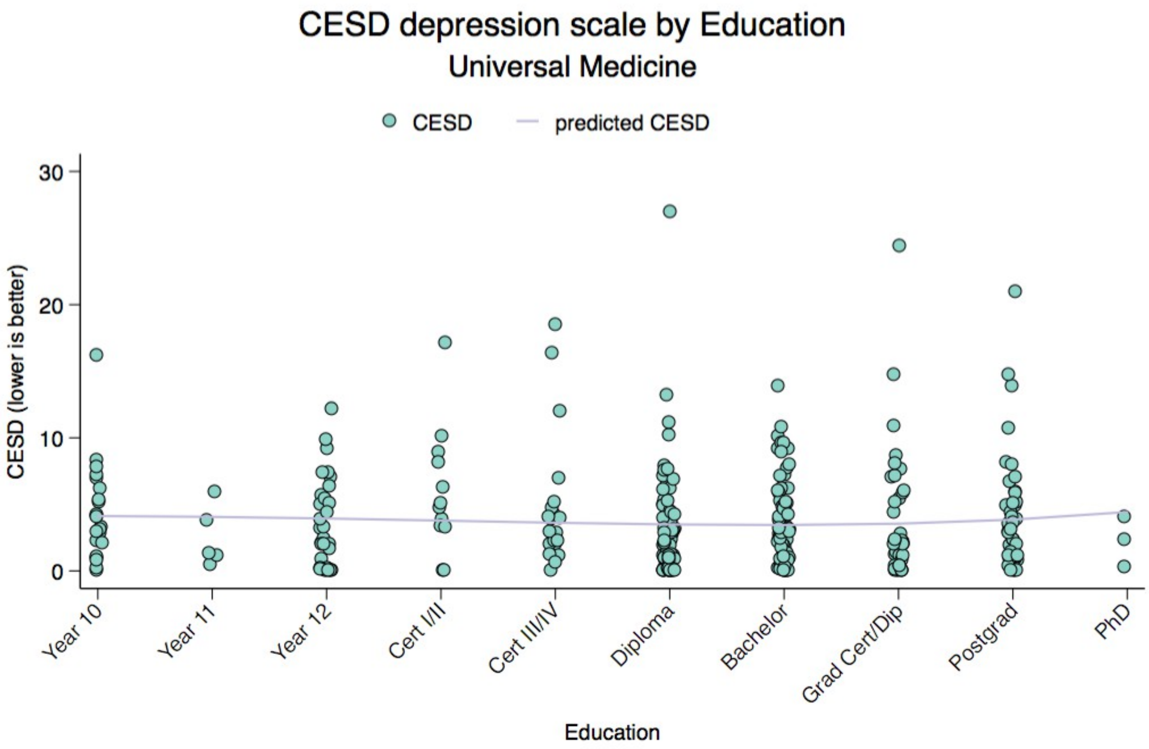

CES-D: Center for Epidemiologic Studies Depression Scale

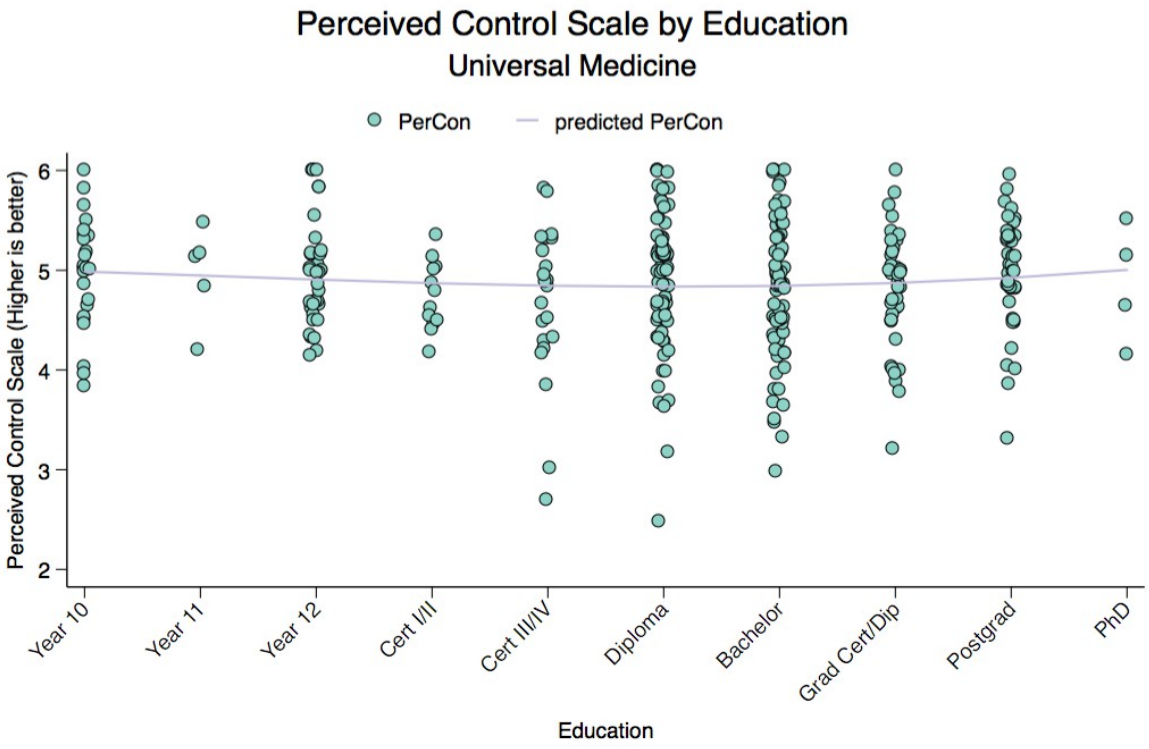

The 'Perceived Control' scale is a scale used by the ALSWH to show how much respondents feel to be in control of their life. This scale was also used in the UM survey and the graph above shows the responses of the UM survey participants.

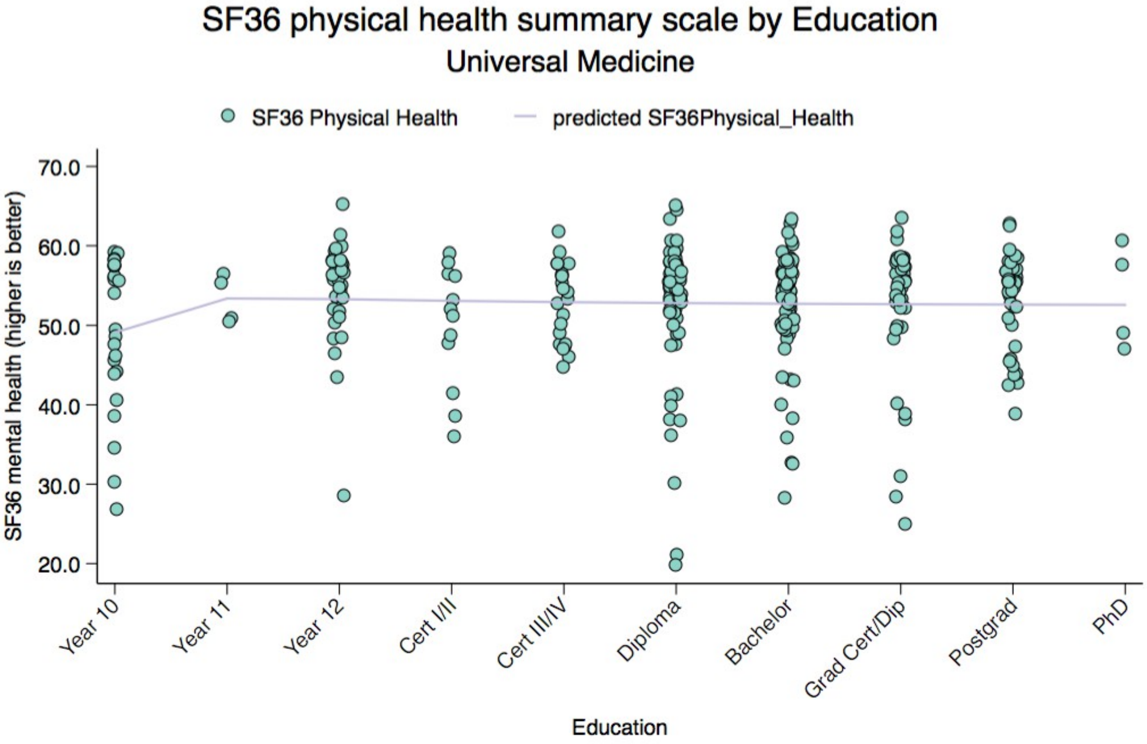

SF-36: 36-Item Short Form Survey. The SF-36 here shows the general physical health of the UM survey respondents. The responses have been scaled to a mean of 50 and a standard deviation of 10.

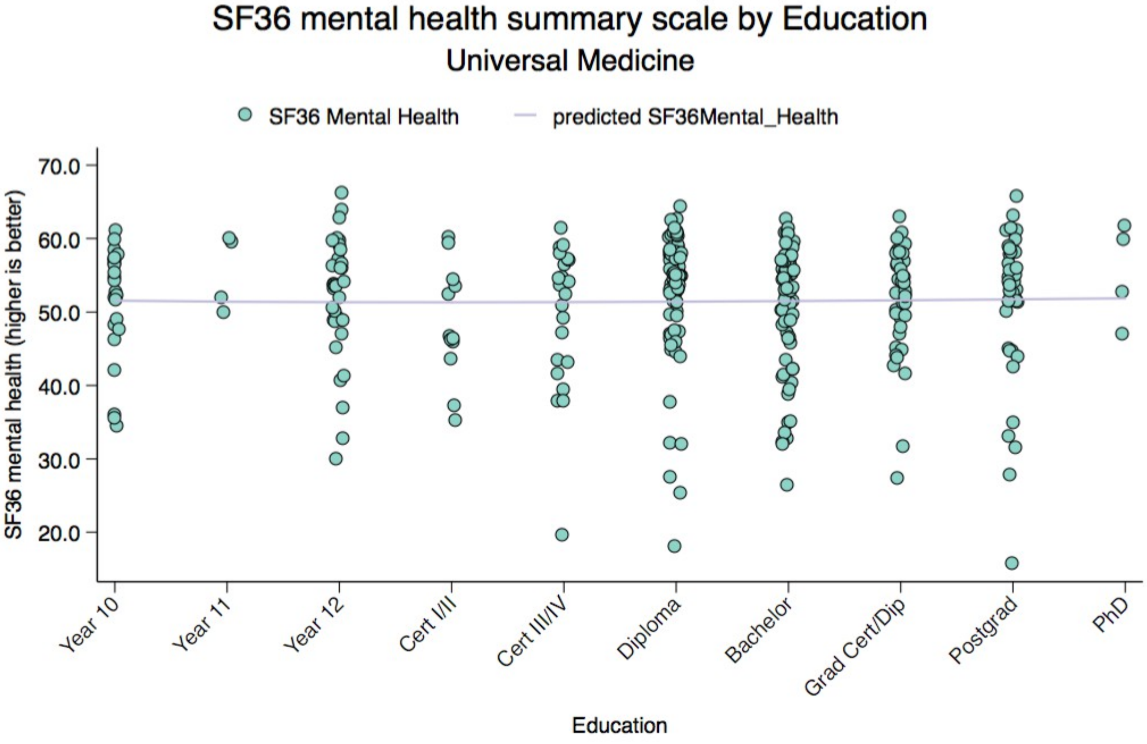

Here the SF-36 shows the general mental health of the respondents.

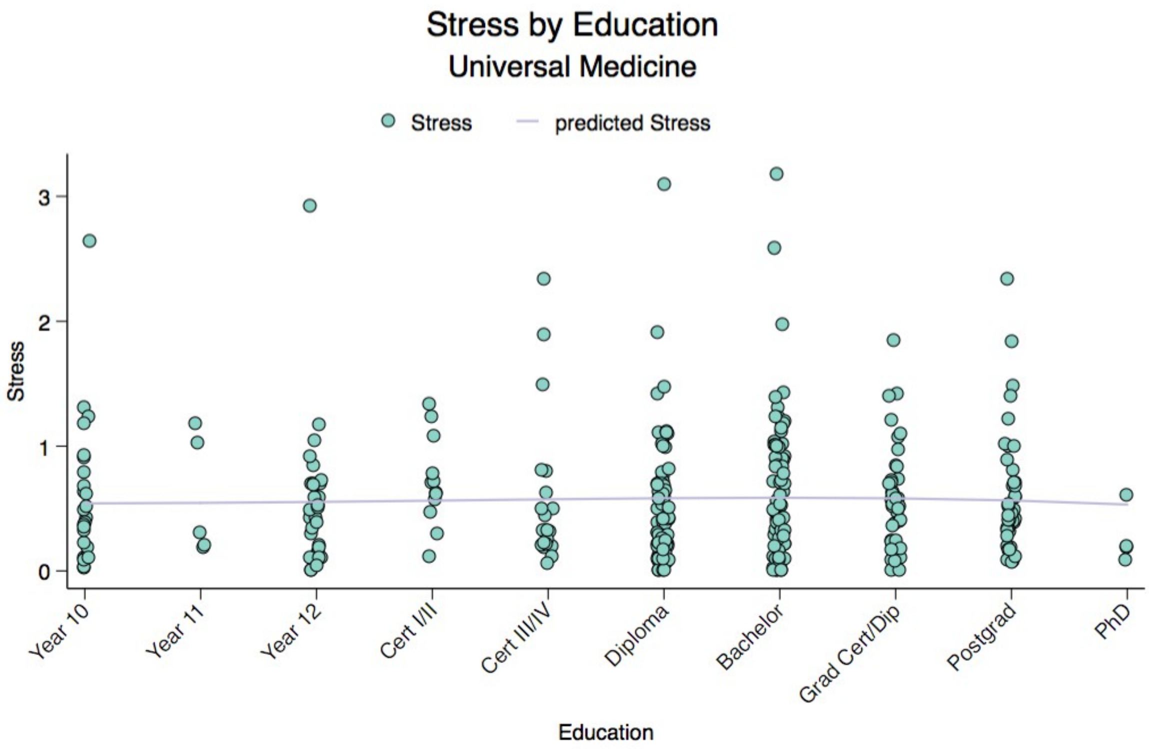

This scale measures the perceived stress of the respondents. Lower numbers are better. As in all other cases the line of best fit is flat, showing no association between education and perceived stress.
